# Psychosocial Risk Factors and Adolescent Problematic Internet Gaming (PIG): The Mediating Roles of Deviant Peer Affiliation and Hedonic Gaming Experience

**DOI:** 10.3390/bs15091177

**Published:** 2025-08-29

**Authors:** Yi Wu, Huazhen Li, Zhanni Luo

**Affiliations:** 1 Research and Advisory Center, Chongqing Normal University, Chongqing 401331, China; 2 College of Education, Health and Human Development, University of Canterbury, Christchurch 8041, New Zealand; 3 School of Foreign Languages and Literatures, Chongqing Normal University, Chongqing 401331, China

**Keywords:** adolescent problematic Internet gaming, gaming addiction, deviant peers, hedonic experience, emotional loneliness

## Abstract

Background: Problematic Internet gaming (PIG), considered an early stage of Internet gaming addiction (IGA), has become increasingly prevalent among adolescents. This study focused on deviant peer affiliation (DPA) and hedonic gaming experience (HGE) as key mediators and examined four psychosocial risk factors closely related to them: interpersonal incompetence (II), perceived stress (PS), frustration (FR), and emotional loneliness (EL). Specifically, the study investigated how these four psychosocial risk factors influence adolescents’ DPA, HGE, and PIG, and whether DPA and HGE mediate these relationships. Methods: Based on existing validated scales, we developed a questionnaire to measure these seven constructs (II, PS, FR, IC, DPA, HGE, and PIG), proposed 14 hypotheses, and collected 214 valid responses from adolescents. Structural equation modeling (SEM) was used to test the hypothesized model. Findings: The results showed that all 14 hypotheses were supported. Specifically, interpersonal incompetence significantly predicted perceived stress; stress led to frustration; and frustration, in turn, contributed to emotional loneliness. Furthermore, all four psychosocial risk factors significantly predicted deviant peer affiliation, hedonic gaming experience, and ultimately, problematic Internet gaming among adolescents. Both DPA and HGE mediated the effects of psychosocial risk factors on adolescent problematic Internet gaming (PIG), with the model explaining moderate-to-high variance. This study highlights the importance of segmenting adolescents into more specific subgroups based on the distinct developmental pathways leading to PIG. Implications: Understanding the step-by-step mechanisms and psychological drivers of different adolescent subtypes can provide a more solid foundation for early identification and targeted intervention efforts.

## 1. Introduction

### 1.1. Internet Gaming Addiction: A Global Health Concern Affecting Adolescents

Playing Internet games is a common form of entertainment. However, excessive engagement can lead to various negative consequences, including strained interpersonal relationships, reduced time for work or study, visual impairments, and increased irritability (Luo & Xie, 2025; Purwaningsih & Nurmala, 2021). When such behavior becomes compulsive and uncontrollable, it can develop into Internet gaming addiction (IGA), which is now widely recognized as a global public health issue (Purwaningsih & Nurmala, 2021).

Adolescents, being at a critical stage of psychological and cognitive development, are particularly susceptible to behavioral addictions (H. Li et al., 2025). Their executive control systems are still maturing, making it more difficult for them to regulate impulses and delay gratification. Moreover, peer influence and the pursuit of social approval further amplify their vulnerability to addictive behaviors (Lam, 2014). These developmental characteristics render adolescents especially at risk for developing Internet gaming addiction.

### 1.2. Shift in Focus: From Addiction to Problematic Use

While Internet gaming addiction represents the more severe end of the behavioral spectrum, research attention has recently shifted toward a broader and earlier phase—problematic Internet gaming (PIG). Internet gaming addiction (IGA) is typically characterized by persistent and uncontrollable Internet gaming that causes significant functional impairment (Kuss & Griffiths, 2012). In contrast, problematic Internet gaming (PIG) refers to a problematic pattern of Internet gaming behavior that does not yet meet the clinical criteria for addiction but may still pose serious risks to adolescents’ well-being (Luo & Xie, 2025).

Scholars have pointed out that by the time individuals meet the diagnostic threshold for addiction, intervention may be more difficult and less effective (Yen et al., 2008). Early identification and prevention at the stage of PIG may therefore offer a more effective strategy for reducing the incidence of gaming addiction (Luo & Xie, 2025). Despite this, the majority of existing research and intervention programs still focus on IGA, while PIG remains relatively underexplored. One possible reason is that individuals who have already developed addiction are more likely to seek clinical treatment, which makes them more accessible as research participants. In addition, the medical community has a stronger research presence, leading to a larger body of studies and publications on clinically diagnosed cases of gaming addiction, while problematic gaming has received comparatively less attention.

Although some findings from IGA research may be applicable to PIG, the unique characteristics of the latter warrant independent investigation. Further research is urgently needed to better understand the psychological and social mechanisms underlying PIG and to inform early intervention efforts.

### 1.3. Psychosocial Risk Factors, Social Adaptation, and Adolescent Problematic Internet Gaming

Psychosocial risk factors refer to a range of psychological and social conditions that increase an individual’s vulnerability to mental health problems and maladaptive behaviors (Baysak et al., 2018; Mohamed et al., 2023; Rho et al., 2018; Simkute et al., 2025). These factors encompass a wide array of individual and contextual challenges, such as low self-esteem, emotional loneliness, perfectionism, social isolation, peer rejection, parental neglect, social marginalization, and economic hardship (Lemmens et al., 2011; H. Li et al., 2025). As they reflect the dynamic interaction between internal vulnerabilities and external stressors, psychosocial risk factors are widely recognized as critical predictors of both the onset and escalation of problematic behaviors, including problematic Internet gaming.

To facilitate more targeted investigations, psychosocial risk factors are often classified into subtypes such as family-related factors (e.g., parental neglect and authoritarian parenting), school-related factors (e.g., academic pressure and school bullying), and interpersonal/relational factors (e.g., social isolation and interpersonal incompetence) (Choo et al., 2015). Difficulties in social adaptation may not only hinder healthy social integration but also contribute to maladaptive coping behaviors, such as excessive or problematic Internet gaming.

In this study, we focus on four psychosocial risk factors closely related to adolescent social adaptation: interpersonal incompetence, perceived stress, frustration, and emotional loneliness. These factors represent a progressive chain of internalized difficulties that often co-occur and reinforce one another. Specifically, adolescents who struggle with interpersonal competence may face repeated failures in social interactions, which heighten their perceived stress in daily life. When such stress is not effectively regulated, it can escalate into frustration—a negative emotional state marked by feelings of helplessness and perceived obstruction of personal goals. This frustration may further contribute to emotional loneliness, characterized by a lack of close emotional bonds and perceived intimacy. This cascade of vulnerabilities can push adolescents to withdraw from real-world social contexts and seek alternative forms of gratification—often in immersive, low-demand environments like online gaming (H. Li et al., 2025; Porat et al., 2025; Puiras et al., 2020; Pyszkowska et al., 2025). Thus, these four interrelated factors provide a theoretically grounded and context-sensitive framework for understanding how deficits in social adaptation may lead adolescents toward problematic Internet gaming.

### 1.4. Deviant Peer Affiliation and Hedonic Gaming Experience: The Mediating Factors

While psychosocial risk factors are fundamental in explaining adolescents’ vulnerabilities to problematic gaming, they alone cannot fully account for the social and motivational processes that transform these vulnerabilities into actual problematic behaviors.

Among the various contributing factors, deviant peer affiliation stands out as particularly significant. Emotionally rejected adolescents are more likely to seek support from deviant peers, and during interactions with these groups, they tend to imitate the maladaptive behaviors exhibited by their peers (Fergusson et al., 2002; Wang et al., 2017). As a result, associating with peers who display excessive gaming tendencies can reinforce an individual’s problematic gaming behavior (Zhu et al., 2017). However, the specific elements that drive individuals to affiliate with deviant peers have yet to be clearly determined.

The hedonic nature of gaming also plays a critical role. Internet games are designed to provide immediate emotional gratification, including excitement, pleasure, and a sense of achievement. For many adolescents, these hedonic rewards are powerful motivators for sustained engagement. While some studies have acknowledged the role of hedonic gaming experiences (Sioni et al., 2017), few have treated them as a central mechanism driving PIG. Most research treats hedonic experience as a secondary outcome, rather than a core factor in the development of problematic Internet gaming. This oversight highlights the need for studies that explicitly examine the affective rewards of gaming as part of the problem’s causal structure.

Therefore, this study conceptualizes deviant peer affiliation and hedonic gaming experience as mediators to elucidate the mechanisms by which psychosocial risk factors influence problematic gaming behaviors.

### 1.5. Research Aims, Research Significance, and Research Questions (RQs)

Building on the existing literature, this study aims to deepen the understanding of adolescent problematic Internet gaming by examining the complex interplay between psychosocial risk factors, social influences, and affective motivations. Specifically, it focuses on four interrelated psychosocial risk factors related to social adaptation— interpersonal incompetence (II), perceived stress (PS), frustration (FR), and emotional loneliness (EL)—and investigates how these factors contribute to problematic gaming behaviors. Furthermore, this study introduces deviant peer affiliation (DPA) and hedonic gaming experience (HGE) as key mediating factors, aiming to clarify the social and emotional mechanisms that translate psychosocial vulnerabilities into problematic Internet gaming.

This research is important because it fills a gap by focusing on problematic Internet gaming, an early stage before full addiction. By looking at both risk factors and their social and emotional pathways, the study offers a clearer picture of how problematic gaming develops.

Ultimately, this study aims to address three research questions (RQs):

RQ1. How do interpersonal incompetence (II), perceived stress (PS), frustration (FR), and emotional loneliness (EL) contribute to adolescents’ deviant peer affiliation (DPA) and hedonic gaming experience (HGE)?

RQ2. What are the relationships among hedonic gaming experience (HGE), deviant peer affiliation (DPA), and adolescents’ problematic Internet gaming (PIG)?

RQ3. Do hedonic gaming experience (HGE) and deviant peer affiliation (DPA) mediate the effects of interpersonal incompetence (II), perceived stress (PS), frustration (FR), and emotional loneliness (EL) on problematic Internet gaming (PIG)?

## 2. Literature Review and Hypotheses

### 2.1. The Sequential Influence of Interpersonal Incompetence (II), Perceived Stress, Frustration (FR), and Emotional Loneliness (EL)

Interpersonal incompetence involves difficulties and feelings of inadequacy in social interactions, often accompanied by negative self-evaluation and diminished social skills (Bhagat et al., 2019; Choi & Choi, 2025). Such difficulties frequently lead to loneliness and increased psychosocial distress, such as anxiety and depression, reinforcing negative perceptions of one’s social abilities. Consequently, individuals may increasingly avoid social situations, further intensifying their sense of isolation.

Within adolescent social networks, interpersonal interactions represent a major source of psychosocial stress, particularly when adolescents encounter negative feedback or rejection from peers. This type of social stress can cause emotional distress, negatively affecting adolescents’ perceptions of their social competence. As a result, adolescents may turn to alternative coping mechanisms, such as online gaming, heightening their risk for Internet gaming addiction (Fiorilli et al., 2019).

Perceived stress is defined as the subjective feeling that environmental demands exceed one’s coping resources, leading to tension, anxiety, and emotional overload (Pera, 2020). Previous research has established that elevated stress levels are associated with emotional disturbances, such as frustration (Zajenkowska et al., 2017). Stress contributes to a sense of helplessness and agitation, which in turn can lead to frustration.

Frustration typically arises when individuals perceive that their efforts to meet goals or desires are hindered. When stress intensifies and the individual feels incapable of managing the stressors effectively, frustration commonly follows. This inability to control stressors and manage their emotional consequences often leads to a state of frustration (Kosa & Uysal, 2021). Thus, the greater the perceived stress, the more intense the experience of frustration, highlighting the gap between individuals’ expectations and their reality.

Frustration in interpersonal contexts—such as repeated failures in initiating or maintaining meaningful social relationships—has been identified as a significant contributor to emotional loneliness. Emotional loneliness refers to the perceived absence of close emotional attachments, such as those found in intimate friendships, romantic partnerships, or supportive familial bonds (DiTommaso et al., 2004). When individuals experience persistent interpersonal difficulties or rejection, they often develop heightened frustration, which can erode their sense of belonging and emotional connectedness (Saricali & Guler, 2022). Such frustration may lead to withdrawal from social engagement or reduced motivation to pursue close relationships, thereby exacerbating feelings of emotional loneliness (Saricali & Guler, 2022).

Therefore, we proposed the following the first three hypotheses:

**H1.**  *Interpersonal incompetence (II) positively predicts perceived stress (PS).*

**H2.**  *Perceived stress (PS) positively predicts frustration (FR).*

**H3.**  *Frustration (FR) positively predicts emotional loneliness (EL).*

### 2.2. Psychosocial Risk Factors and Deviant Peer Affiliation (DPA)

Deviant peer affiliation (DPA) refers to the selective association with peers involved in delinquent behaviors such as substance abuse and aggression, and is increasingly recognized as a pivotal issue in adolescent behavioral studies (Wang et al., 2017). According to the Ecological Systems Theory, adolescents lacking positive peer relationships or experiencing interpersonal difficulties tend to affiliate with deviant peers to gain acceptance (Bronfenbrenner, 1986). Prior research consistently indicates that interpersonal incompetence, perceived stress, frustration, and identity crises significantly predict adolescents’ deviant peer affiliations (Mrug et al., 2012). Thus, we proposed H4 to H7:

**H4.**  *Interpersonal incompetence (II) positively leads to deviant peer affiliation (DPA).*

**H5.**  *Perceived stress (PS) positively leads to deviant peer affiliation (DPA).*

**H6.**  *Frustration (FR) positively leads to deviant peer affiliation (DPA).*

**H7.**  *Emotional loneliness (EL) positively leads to deviant peer affiliation (DPA).*

### 2.3. Psychosocial Risk Factors and Hedonic Gaming Experience (HGE)

Real-world stressors such as interpersonal conflicts, perceived stress, frustration, and identity crises compel adolescents to seek emotional relief through online gaming. This behavior, termed “game escapism”, provides adolescents with temporary enjoyment, competence, social connection, and self-validation (Billieux et al., 2013; Kaczmarek & Drazkowski, 2014). By fulfilling unmet psychological needs and alleviating negative emotions, online gaming becomes an appealing coping strategy (Blinka & Mikuška, 2014). Thus, perceived stress, interpersonal conflicts, frustration, and emotional loneliness specifically lead adolescents toward gaming as a coping mechanism. These experiences promote hedonic gaming experience, characterized by emotional relief and pleasure derived from gaming (Caplan et al., 2009).

Therefore, we proposed the following hypotheses:

**H8.**  *Interpersonal incompetence (II) positively predicts hedonic gaming experience (HGE).*

**H9.**  *Perceived stress (PS) positively predicts hedonic gaming experience (HGE).*

**H10.**  *Frustration (FR) leads to hedonic gaming experience (HGE).*

**H11.**  *Emotional loneliness (EL) leads to hedonic gaming experience (HGE).*

### 2.4. Deviant Peer Affiliation, Hedonic Gaming Experience, and Adolescent Problematic Internet Gaming

In addition to individual psychological vulnerabilities, both deviant peer affiliation (DPA) and hedonic gaming experience (HGE) play critical roles in shaping adolescents’ engagement in problematic Internet gaming.

Research suggests that hedonic gaming experiences may not only fulfill emotional needs but also facilitate social bonding with peers who exhibit similar gaming behaviors, thereby increasing the likelihood of affiliation with deviant peer groups (S. Li et al., 2022). Accordingly, the following hypothesis is proposed:

**H12.**  *Hedonic gaming experience (HGE) positively predicts deviant peer affiliation (DPA).*

Adolescents experiencing peer rejection often turn to deviant peers, who engage in high-risk behaviors such as excessive gaming, further isolating them from adaptive peer groups. This isolation increases their vulnerability to problematic Internet gaming. Peer-rejected adolescents frequently form relationships with similarly rejected peers, amplifying engagement in maladaptive behaviors including problematic Internet gaming (Wang et al., 2017). Adolescents with higher sensation-seeking tendencies are especially vulnerable to associating with deviant peers under stressful settings, promoting problematic gaming practices (S. Li et al., 2022). Furthermore, stressful school environments can drive adolescents towards deviant peer groups, intensifying their reliance on gaming as a coping strategy (Hussain et al., 2012).

Additionally, according to the Hedonic Management Model, adolescents may become addicted to activities like gaming due to their ability to induce pleasure or relieve negative emotions. Positive gaming experiences, such as feelings of achievement and success, reinforce self-identity and emotional well-being, thus increasing dependence on games (Z. W. Y. Lee et al., 2020). Moreover, negative reinforcement occurs as adolescents use gaming to escape from daily stressors, perpetuating problematic gaming behaviors (Hussain et al., 2012). Thus, hedonic gaming experiences significantly contribute to adolescent problematic Internet gaming. Therefore, we proposed the following hypotheses:

**H13.**  *Deviant peer affiliation (DPA) positively leads to adolescent problematic Internet gaming (PIG).*

**H14.**  *Hedonic gaming experience (HGE) positively predicts adolescent problematic Internet gaming (PIG).*

**Figure 1:**
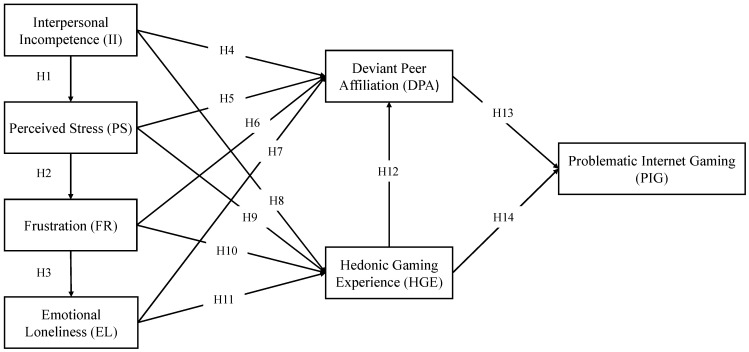
Theoretical framework and hypotheses.

The theoretical framework and hypotheses are depicted in Figure 1.

## 3. Methods

### 3.1. Research Instruments

The questionnaire used in this study had two main sections. The first section gathered demographic information from participants. This included their gender and current grade level. The second section featured seven subscales. All of these subscales were adapted from previously established and validated measures (see Supplementary Materials).

The subscale measuring interpersonal incompetence (II) was adapted from the Social Self-Efficacy subscale of the Self-Efficacy Scale developed by Sherer et al. (1982). Although the original scale was designed to assess individuals’ self-efficacy in handling social situations, many of its items reflect observable behaviors and tendencies that are also indicative of interpersonal functioning. Namely, we believe that despite the terminological shift from “social” to “interpersonal”, the item content remains highly relevant to the current research context. The adapted items include “I have difficulty communicating with others” and “I don’t feel confident while initiating the conversation with others.” To ensure conceptual and contextual validity, the adapted items were reviewed by two domain experts specializing in adolescent development and social competence. They assessed both the face validity—whether the items appeared to measure interpersonal incompetence from a respondent’s perspective—and the content relevance of each item within the adolescent population. The adapted scale was also found to have strong internal consistency, with full psychometric details reported in the Section 4.

The subscale measuring perceived stress (PS) was adapted from the 14-item Perceived Stress Scale (PSS) developed by Cohen et al. (1983). The original PSS was designed to measure the degree to which situations in one’s life are appraised as stressful, unpredictable, uncontrollable, and overloading, asking about feelings and thoughts during the last month (e.g., “In the last month, how often have you been upset because of something that happened unexpectedly?”). This study adapted five items, such as “How often have you been angry about things you can’t control?”

The subscale measuring frustration (FR) drew from concepts in the Basic Psychological Need Satisfaction and Frustration Scale, informed by work such as Kosa and Uysal (2021) and Allen and Anderson (2018). Kosa and Uysal (2021) investigated need frustration in online video games, with their frustration subscales measuring autonomy frustration (e.g., “I feel forced to do many things I wouldn’t choose to do in [my current favorite online game].”), competence frustration, and relatedness frustration. Allen and Anderson (2018) also assessed need frustration in both the real world and video games. Our adapted subscale used five items reflecting autonomy and competence frustration. Example items include “I feel that I am unable to control the important things in my life.”

The subscale measuring emotional loneliness (EL) was adapted from the Social and Emotional Loneliness Scale for Adults (SELSA) by DiTommaso et al. (2004). The original 37-item SELSA is a multidimensional measure of emotional (romantic and family) and social loneliness. In this process, we paid particular attention to removing survey items related to adults and selecting those relevant to our target population: adolescents. We adapted five items to reflect aspects such as a lack of social connectedness. For instance, one item states: “I don’t have any friends who share my views, but I wish I did.”

The deviant peer affiliation (DPA) subscale was adapted from the deviant peers questionnaire used by Zhu et al. (2017), which asked respondents “to indicate how many of their friends during the past 6 months displayed deviant behaviors (e.g., How many of your friends got involved in fights during the past 6 months?)”. Foundational work by Fergusson et al. (2002) also explored the influence of deviant peer affiliations on crime and substance use. Our subscale measures how often adolescents associate with peers involved in problematic activities. An example item is “How many of your friends have cheated on exams in the past six months?”

The hedonic gaming experience (HGE) subscale was based on the existing literature. For instance, Chang et al. (2014) explored hedonic outcome expectations in online games, defining them as “expectations associated with online game playing that may result in different forms of enjoyment (such as playfulness, fun and pleasure)”. Liu and Li (2011) also studied mobile hedonic services, including perceived enjoyment in mobile gaming. Our subscale measures the satisfaction adolescents gain from Internet games. An example is “I think the process of playing Internet games would be pleasant.”

Finally, the subscale for adolescent problematic Internet gaming (PIG) was adapted from the Game Addiction Scale (GAS) developed by Lemmens et al. (2009). The original 21-item GAS was designed for adolescents and measures seven criteria including salience (e.g., “Did you think about playing a game all day long?”), tolerance, mood modification, relapse, withdrawal, conflict, and problems. Our adapted version included five items. One example is: “I have felt depressed or irritated when I cannot play internet video games.”

In this study, most subscales used a 5-point Likert scale, ranging from 1 (strongly disagree) to 5 (strongly agree), to assess participants’ agreement with the items. However, the deviant peer affiliation (DPA) subscale used a different response format. Instead of scoring their agreement on a Likert scale, participants for the DPA questions were asked to identify the number of their friends who engaged in various behaviors in the last six months.

Each subscale demonstrated satisfactory internal reliability, with Cronbach’s alpha values ranging from 0.878 to 0.916. In addition, exploratory factor analysis (EFA) was conducted to examine the overall structure of the questionnaire and to ensure the construct validity of the scale. The detailed findings will be presented in the Section 4.

### 3.2. Data Collection, Participants, and Ethics Considerations

After finalizing the questionnaire, we hired a professional translator to translate it into Chinese. To ensure accuracy, we used the back-translation method. Once the translation was complete, we converted the questionnaire into a digital format. The surveys were subsequently administered to adolescents in Southwest China using a convenience sampling method. The surveys were collected both electronically and in paper form. Data collection lasted for one month.

In total, we collected 247 surveys. However, 33 responses failed the attention-check questions. Therefore, we ended up with 214 valid responses. According to Hair et al. (2021), a sample size between 100 and 200 is generally considered sufficient for models of moderate complexity (i.e., those involving 3 to 7 latent variables and 10 to 20 structural paths) when using PLS-SEM. The final sample of 214 valid responses exceeds the recommended threshold and is considered adequate for robust model estimation.

Among these 214 participants, the age range was 13–16. The number of male (n = 102, 47.7%) and female (n = 112, 52.3%) participants was roughly equal. There were 54 junior high school students (25.2%) and 160 senior high school students (74.8%). All participants were aware of the research intentions and signed the consent form. Participation was completely anonymous and voluntary.

### 3.3. Data Analysis

This study adopted a structural equation modeling (SEM) approach to examine the psychosocial risk factors and mediating factors underlying adolescent problematic Internet gaming (PIG). We employed both SPSS 26.0 and SmartPLS 4.0 to conduct a comprehensive analysis of the survey data collected from 214 adolescent participants.

Firstly, we conducted a descriptive analysis to calculate the means and standard deviations of each construct. Next, we performed a reliability analysis that tested the Cronbach’s alpha of the scale. Then, we examined the convergent validity and discriminant validity of the proposed model. The results indicated that the internal consistency of each subscale and the relationships between the subscales were acceptable for structural equation modeling tests. Finally, we used SmartPLS to test the fit index of the two models and the 14 hypotheses raised.

## 4. Results

### 4.1. Reliability and Validity Analysis

The dataset’s suitability for factor analysis was verified. The Kaiser–Meyer–Olkin (KMO) measure reached 0.939, which is well above the commonly accepted cutoff of 0.60, indicating excellent sampling adequacy. Bartlett’s Test of Sphericity also yielded a chi-square value of 5530.594 (*p* < 0.001), confirming sufficient correlations among variables. Moreover, all items’ communalities (extraction) were greater than the commonly recommended threshold of 0.40, providing further evidence of their appropriateness for factor analysis.

Subsequently, Principal Axis Factoring with a Direct Oblimin rotation was conducted, and seven factors were extracted according to the eigenvalue-greater-than-one rule. The results show that all survey items have factor loadings ranging from 0.522 to 0.823, demonstrating strong associations with their respective constructs (see Table 1). Together, these components explain 69.693% of the total variance, indicating a satisfactory model fit and the effectiveness of the constructs in accounting for data variability (Härdle et al., 2017).

The reliability and validity are further supported by high Cronbach’s alpha coefficients (0.872 to 0.929), indicating strong internal consistency. As shown in the table, most constructs achieved AVE values above the recommended threshold of 0.50; for those with AVE values between 0.40 and 0.50, the convergent validity is still considered acceptable because all CR values exceed 0.70 across the seven factors (Qu, 2007).

Table 2 shows the correlation matrix and square root of the AVE, which are used to measure the discriminant validity. The square roots of the AVE are higher than the inter-correlations between constructs, meeting the Fornell and Larcker (1981) criteria. These results imply moderate to strong construct validity. However, some constructs may need to be refined.

Significant correlations (*p* < 0.01) among constructs demonstrate key relationships influencing adolescent behavior. Perceived stress (PS) shows a notable positive correlation with deviant peer affiliation (DPA) (*r* = 0.582), suggesting that adolescents experiencing higher stress levels may become more inclined to affiliate with deviant peers. Additionally, emotional loneliness (EL) is significantly correlated with interpersonal incompetence (II) (*r* = 0.506). These correlations underline how emotional and psychological pressures can significantly shape adolescents’ social interactions and behaviors.

Moreover, adolescent problematic Internet gaming (PIG) suggests a strong association with DPA (*r* = 0.564) and hedonic gaming experience (*r* = 0.560), indicating that problematic gaming behaviors may be significantly influenced by both peer relationships and enjoyment of gaming. Furthermore, the correlation between PIG and perceived stress (*r* = 0.314) suggests gaming may serve as a coping mechanism for adolescents facing stressful conditions. Despite these substantial correlations, the discriminant validity across constructs remains robust, confirming the structural integrity of the theoretical model applied.

### 4.2. Model Fit and Hypothesis Testing

The data was analyzed using SmartPLS 4.0, and the Variance Inflation Factor (VIF) values for all latent variables were determined to be less than two, indicating that the model does not contain severe multicollinearity and that the latent variables are highly independent. The low VIF values indicate that the linear connection among latent variables is limited, preventing difficulties like unstable path coefficients or estimation bias due to multicollinearity.

This finding reinforces the structural equation model’s reliability, laying the groundwork for future path analysis and hypothesis testing. It also shows that the dataset is of good quality, with the relationships between latent variables faithfully reflecting the theoretical model’s assumptions. As a result, the study’s explanatory power and scientific rigor increase.

The results of the path analysis provide robust support for the hypothesized relationships among interpersonal incompetence, perceived stress, frustration, and emotional loneliness. Specifically, interpersonal incompetence significantly predicted perceived stress (*β* = 0.383, *t* = 6.631, *p* < 0.001), confirming H1. In turn, perceived stress positively influenced frustration (*β* = 0.470, *t* = 8.236, *p* < 0.001), supporting H2. Moreover, frustration was found to be a significant predictor of emotional loneliness *(β* = 0.460, *t* = 8.569, *p* < 0.001), thus validating H3 (see Table 3).

**Figure 2:**
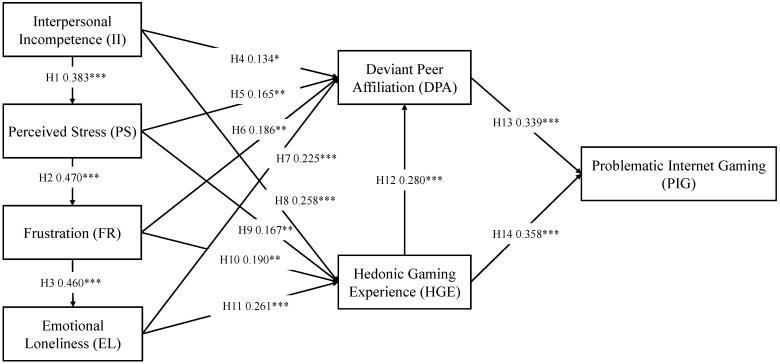
The hypothesis-testing result (*p* < 0.05 = *, *p* < 0.01 = **, *p* < 0.001 = ***).

The analysis further looked at what factors might lead to deviant peer affiliation. Interpersonal incompetence had a small but meaningful impact on deviant peer affiliation (*β* = 0.134, *t* = 2.590, *p* = 0.010), which supports H4. Perceived stress also had a significant effect (*β* = 0.165, *t* = 2.804, *p* = 0.005). Similarly, frustration showed a positive relationship with DPA (*β* = 0.186, *t* = 2.962, *p* = 0.003), and so did emotional loneliness (*β* = 0.225, *t* = 3.533, *p* < 0.001). These findings support H5, H6, and H7. Overall, the results suggest that when adolescents feel stressed or emotionally overwhelmed, they may turn to deviant peers. This could be a way of coping, even if it leads to negative outcomes. The findings are in line with existing theories that highlight how social environments can shape behavior, especially when young people are under internal pressure (see Figure 2).

The model’s final section investigated how the hedonic gaming experience (HGE) influences problematic gaming. It also investigated if hedonic gaming experience serves as a link between emotional elements and gaming behavior. The results indicated that all four variables, interpersonal incompetence, perceived stress, frustration, and emotional loneliness, were positively and significantly linked to hedonic gaming experience (HGE). These findings supported H8, H9, H10, and H11. The positive connections were statistically significant (*p* < 0.01) and had standardized path coefficients ranging from 0.167 to 0.261. This suggests that adolescents may turn to gaming as a way to handle emotional discomfort.

The analysis also showed that both hedonic gaming experience (HGE) (*β* = 0.358, *t* = 5.193, *p* < 0.001) and deviant peer affiliation (DPA) (*β* = 0.339, *t* = 5.177, *p* < 0.001) had strong and significant effects on adolescent problematic Internet gaming (PIG). These results confirmed H13 and H14. Taken together, the findings show that emotional difficulties and peer influence both play important roles in the development of problematic gaming behavior.

### 4.3. Prediction Power of the Variables in the Study

The *R*-square values indicate the model’s explanatory power. The adjusted *R*-square values for all variables ranged from 0.184 to 0.587, indicating that the model possesses moderate to high explanatory power. This suggests that the proposed constructs effectively capture key factors affecting adolescent problematic Internet gaming. However, there is still room to add more variables or explore moderating effects.

## 5. Discussion

The current study investigated the pathways to adolescent problematic Internet gaming, considering psychosocial risk factors, deviant peer affiliation (DPA), and hedonic gaming experience (HGE). Path analysis strongly supported the proposed theoretical model, confirming all 14 hypotheses. The model showed moderate-to-high explanatory power, notably for DPA (*R*^2^ adjusted = 0.587) and HGE (*R*^2^ adjusted = 0.442), and moderate power for PIG (*R*^2^ adjusted = 0.384).

The analysis confirmed Hypotheses 1, 2, and 3: interpersonal incompetence (II) significantly predicted perceived stress (PS); PS, in turn, influenced frustration (FR); and FR predicted emotional loneliness. These findings align with existing theories suggesting that social difficulties contribute to increased stress (Bhagat et al., 2019), stress fosters frustration (Pera, 2020), and frustration brings emotional loneliness (Zajenkowska et al., 2017). Collectively, this pathway underscores how challenges in social adaptation—reflected in poor interpersonal functioning, heightened sensitivity to social feedback, and inadequate coping resources—can accumulate and manifest as deeper psychological vulnerabilities. Such psychosocial risk factors not only compromise emotional well-being but also disrupt the formation of a stable and coherent identity during adolescence.

Following this internal progression, these psychosocial risk factors were found to push adolescents towards deviant peers. Interpersonal incompetence, perceived stress, frustration, and emotional loneliness all positively predicted deviant peer affiliation (DPA) (H4–H7). This is consistent with Bronfenbrenner’s Ecological Systems Theory (Bronfenbrenner, 1986) and research showing distress can lead adolescents to seek acceptance in deviant groups (Zhu et al., 2017). Thus, adolescents who feel inadequate, stressed, or uncertain about their identity may be more inclined to seek group membership, even when such affiliations reinforce problematic social behaviors.

Psychosocial risk factors also predicted better hedonic gaming experience (HGE) (H8–H11). This supports the idea of “game escapism” (Billieux et al., 2013). From a stress-coping perspective, adolescents experiencing internal struggles may use gaming to regulate emotions, relieve stress, and fulfill unmet social or emotional needs. Empirical studies have similarly found that higher levels of stress or emotional loneliness are associated with increased engagement in gaming for pleasure and escape, which may inadvertently reinforce problematic gaming patterns (J.-y. Lee et al., 2019; Yilmaz & Tunca, 2024).

Finally, the analysis focused on how deviant peer affiliation and hedonic gaming experience directly predict problematic Internet gaming. The findings confirmed crucial roles for both. Deviant peer affiliation (DPA) and hedonic gaming experience (HGE) strongly and positively predicted PIG (H13 and H14). Additionally, we also found that HGE positively predicted DPA (H12). This shows an interaction in which good gaming experiences may not only feed problematic usage but also allow relationships with deviant peers, reinforcing both paths. Overall, the findings clearly show that enjoyment from gaming and the influence of deviant peers are related factors in the development and maintenance of PIG.

A large number of previous studies have employed structural equation modeling (SEM) techniques to examine the relationships among multiple factors associated with Internet gaming addiction (IGA). For example, Zhu et al. (2015) used SEM techniques to investigate how parental, school, and peer influences contribute to IGA among early adolescents. Their findings showed that both school connectedness and deviant peer affiliation fully mediated the relationship between parent–adolescent interaction and adolescent gaming addiction. Similarly, J.-y. Lee et al. (2019) conducted an SEM study among South Korean college students to examine the roles of loneliness, regulatory focus, and interpersonal competence in online game addiction, concluding that both loneliness and poor interpersonal competence significantly predicted addictive gaming behaviors. Other studies have also employed structural equation modeling (SEM) to examine the relationships between various factors and adolescent Internet gaming addiction, including but not limited to self-efficacy, achievement, curiosity, and flow (Yilmaz & Tunca, 2024). However, these studies often examine a wide range of variables without a unified theoretical framework, making it difficult to draw cohesive conclusions or identify core drivers of gaming-related problems.

It is noteworthy that the present study was designed to examine whether the four psychosocial risk factors influence deviant peer affiliation (DPA) and hedonic gaming experience (HGE) as mediators, and whether these mediators, in turn, contribute to adolescents’ problematic Internet gaming (PIG). This framework reflects a unidirectional process. However, in reality, the relationships may not be strictly one-way. For instance, deviant peer affiliation could further exacerbate adolescents’ interpersonal incompetence, while experiencing high levels of hedonic gratification in gaming may, paradoxically, intensify frustration or emotional loneliness in their real lives. These potential reciprocal and bidirectional dynamics warrant further investigation in future longitudinal or experimental studies.

At the same time, it should be acknowledged that numerous factors may contribute to adolescents’ problematic Internet gaming (PIG), with family-related variables often playing a central role. For example, parental phubbing, parental conflicts, and parental neglect have been shown to increase adolescents’ vulnerability to problematic gaming behaviors (H. Li et al., 2025). Nevertheless, the present study deliberately focused on deviant peer affiliation (DPA) and hedonic gaming experience (HGE), as well as the individual-level psychosocial factors that are closely linked to these two mediators. This focus was chosen to better capture the peer- and motivation-related mechanisms underlying adolescents’ gaming behaviors, while recognizing that family dynamics remain an essential direction for future research.

It is also worth noting that the majority of existing research tends to focus on Internet gaming addiction (IGA), while less attention has been paid to problematic Internet gaming (PIG). One possible reason is that individuals with clinical-level addiction are more likely to seek medical help, making them more accessible to researchers in clinical settings. In contrast, PIG—though not yet reaching the threshold of clinical impairment—remains under-researched, particularly outside of medical disciplines (Luo & Xie, 2025). However, this does not imply that PIG is unimportant. On the contrary, studying PIG may offer greater practical value for early detection and prevention, as it allows for intervention before the behavior escalates into full-blown addiction (Luo & Xie, 2025).

## 6. Conclusions

### 6.1. Summary

This study explored the relationships among interpersonal incompetence (II), perceived stress (PS), frustration (FR), emotional loneliness (EL), deviant peer affiliation (DPA), hedonic gaming experience (HGE), and adolescent problematic internet gaming (PIG). The findings demonstrated clear links between personal challenges and increased affiliation with deviant peers, as well as heightened enjoyment from gaming experiences. Furthermore, both deviant peer affiliation and pleasurable gaming experiences were positively associated with problematic gaming behaviors. Notably, enjoyable gaming experiences also strengthened connections with deviant peers, highlighting the complex interaction between emotional rewards from gaming and negative peer influences.

### 6.2. Implications

These findings offer practical guidance for professionals and policymakers addressing problematic Internet gaming in adolescents. The study emphasizes the importance of strengthening interpersonal skills and effectively managing emotional distress, which are key targets in many interventions (Zajac et al., 2017). Schools and community organizations should develop targeted programs promoting healthier social interactions and effective stress management techniques. Incorporating emotional education and healthier coping strategies into school curricula may also help adolescents identify alternatives to gaming for mood regulation (Luo, 2021). Implementing these strategies could reduce adolescents’ dependence on gaming for emotional regulation and weaken problematic peer bonds.

### 6.3. Limitations and Future Work

Despite these insights, the study has several limitations that suggest areas for future research. First, the use of structural equation modeling (SEM) relies on cross-sectional data, which restricts the ability to make strong causal inferences. Future studies should employ longitudinal designs to clarify these dynamics over time. Second, the specific demographic characteristics of the sample may limit the generalizability of the results. Replicating the study across diverse cultural and socioeconomic groups could address this issue. Finally, including additional factors, such as parental influences or specific game features, could further enrich the model and enhance its predictive capability. Given the important role of emotional processes, adolescents’ difficulties in emotion regulation could also be considered in future models as a potential contributing factor (Yayla et al., 2025). In addition, the theoretical justification for the mediating effects of deviant peer affiliation and hedonic gaming experience could be further developed in future research.

Future research should also adopt a more targeted approach by segmenting adolescent populations into specific subgroups. For instance, adolescents with pronounced interpersonal difficulties may follow a unique developmental trajectory shaped by particular environmental contexts, psychological processes, and mediating mechanisms that contribute to Internet gaming addiction or problematic Internet gaming (PIG). Uncovering these subgroup-specific pathways could help pinpoint the most influential and modifiable psychosocial factors, thereby enhancing the effectiveness of early detection and intervention.

## Figures and Tables

**Table 1:** Survey items and the exploratory factor analysis.

Construct	Item Code	Factor/Factor Loadings						
		1	2	3	4	5	6	7
Interpersonal Incompetence (II)	II1		0.823					
	II2		0.727					
	II3		0.698					
	II4		0.761					
	II5		0.822					
Perceived Stress (PS)	PS1				0.676			
	PS2				0.717			
	PS3				0.756			
	PS4				0.724			
	PS5				0.684			
Frustration (FR)	FR1			0.682				
	FR2			0.794				
	FR3			0.728				
	FR4			0.776				
	FR5			0.701				
Emotional Loneliness (EL)	EL1						−0.611	
	EL2						−0.661	
	EL3						−0.688	
	EL4						−0.667	
	EL5						−0.836	
Deviant Peer Affiliation (DPA)	DPA1	0.578						
	DPA2	0.619						
	DPA3	0.600						
	DPA4	0.784						
	DPA5	0.541						
	DPA6	0.627						
	DPA7	0.772						
	DPA8	0.646						
Hedonic Gaming Experience (HGE)	HGE1					0.690		
	HGE2					0.780		
	HGE3					0.522		
	HGE4					0.765		
	HGE5					0.542		
Adolescent Problematic Internet Gaming (PIG)	PIG1							0.724
	PIG2							0.800
	PIG3							0.802
	PIG4							0.757
	PIG5							0.729
Cronbach’s Alpha (α):		0.929	0.901	0.877	0.872	0.883	0.890	0.897
AVE (Average Variance Extracted):		0.424	0.590	0.544	0.507	0.447	0.485	0.582
CR (Composite Reliability):		0.853	0.877	0.856	0.837	0.797	0.823	0.874

**Table 2:** Correlation and the square root of the AVE **(in bold)**.

Construct	AVE	II	PS	FR	EL	DPA	HGE	API
Interpersonal Incompetence (II)	0.590	**0.7** **68**						
Perceived Stress (PS)	0.507	0.430 **	**0.7** **12**					
Frustration (FR)	0.544	0.216 **	0.489 **	**0.7** **38**				
Emotional Loneliness (EL)	0.485	0.506 **	0.505 **	0.495 **	**0.** **696**			
Deviant Peer Affiliation (DPA)	0.424	0.522 **	0.582 **	0.533 **	0.647	**0.6** **51**		
Hedonic Gaming Experience (HGE)	0.447	0.490 **	0.500 **	0.470 **	0.558 **	0.630 **	**0.** **669**	
Adolescent Problematic Internet Gaming (PIG)	0.582	0.226 **	0.314 **	0.403 **	0.307 **	0.564 **	0.560 **	**0.7** **63**

Note: The table shows the square root of the AVE on the diagonal and correlations between the latent constructs on the off-diagonal. ** *p* < 0.01.

**Table 3:** Hypotheses-testing results.

Code	Path	O	*T*-Value	*p*-Value	Hypothesis	Result	Hypothesis-Testing Result
H1	II → PS	0.383	6.631	***	Positive	Positive	Confirmed
H2	PS → FR	0.470	8.236	***	Positive	Positive	Confirmed
H3	FR → EL	0.460	8.569	***	Positive	Positive	Confirmed
H4	II → DPA	0.134	2.590	*	Positive	Positive	Confirmed
H5	PS → DPA	0.165	2.804	**	Positive	Positive	Confirmed
H6	FR → DPA	0.186	2.962	**	Positive	Positive	Confirmed
H7	EL → DPA	0.225	3.533	***	Positive	Positive	Confirmed
H8	II → HGE	0.258	4.143	***	Positive	Positive	Confirmed
H9	PS → HGE	0.167	2.634	**	Positive	Positive	Confirmed
H10	FR → HGE	0.190	2.850	**	Positive	Positive	Confirmed
H11	EL → HGE	0.261	3.661	***	Positive	Positive	Confirmed
H12	HGE → DPA	0.280	4.675	***	Positive	Positive	Confirmed
H13	DPA → PIG	0.339	5.177	***	Positive	Positive	Confirmed
H14	HGE → PIG	0.358	5.193	***	Positive	Positive	Confirmed

Note: O = standardized path coefficient; T-value = T statistic for significance testing; *p*-value = significance level (*p* < 0.05 = *, *p* < 0.01 = **, *p* < 0.001 = ***).

## Data Availability

The dataset is publicly available on Figshare at the following link: https://doi.org/10.6084/m9.figshare.29487155.v1.

## Supplementary Materials

The survey is publicly available on Figshare at the following link: https://doi.org/10.6084/m9.figshare.29486420.v1.

## Abbreviations

The following abbreviations are used in this manuscript:

II	Interpersonal incompetence
PS	Perceived stress
FR	Frustration
EL	Emotional loneliness
DPA	Deviant peer affiliation
HGE	Hedonic gaming experience
PIG	Problematic Internet gaming
